# Structural and functional similarities and differences in nucleolar Pumilio RNA-binding proteins between Arabidopsis and the charophyte *Chara corallina*

**DOI:** 10.1186/s12870-020-02444-x

**Published:** 2020-05-24

**Authors:** Su Hyun Park, Hyung-Sae Kim, Prakash Jyoti Kalita, Sang-Bong Choi

**Affiliations:** Division of Bioscience and Bioinformatics, Myongji University, Yongin, Kyunggi-do 449-728 South Korea

**Keywords:** *Arabidopsis thaliana*, *Chara corallina*, Charophyta, ITS2, Puf, RNA-binding proteins, rRNA

## Abstract

**Background:**

Pumilio RNA-binding proteins are evolutionarily conserved throughout eukaryotes and are involved in RNA decay, transport, and translation repression in the cytoplasm. Although a majority of Pumilio proteins function in the cytoplasm, two nucleolar forms have been reported to have a function in rRNA processing in Arabidopsis. The species of the genus *Chara* have been known to be most closely related to land plants, as they share several characteristics with modern Embryophyta.

**Results:**

In this study, we identified two putative nucleolar Pumilio protein genes, namely, *ChPUM2* and *ChPUM3*, from the transcriptome of *Chara corallina*. Of the two ChPUM proteins, ChPUM2 was most similar in amino acid sequence (27% identity and 45% homology) and predicted protein structure to Arabidopsis APUM23, while ChPUM3 was similar to APUM24 (35% identity and 54% homology). The transient expression of *35S:ChPUM2-RFP* and *35S:ChPUM3-RFP* showed nucleolar localization of fusion proteins in tobacco leaf cells, similar to the expression of *35S:APUM23-GFP* and *35S:APUM24-GFP*. Moreover, *35S:ChPUM2* complemented the morphological defects of the *apum23* phenotypes but not those of *apum24,* while *35S:ChPUM3* could not complement the *apum23* and *apum24* mutants. Similarly, the *35S:ChPUM2/apum23* plants rescued the pre-rRNA processing defect of *apum23*, but *35S:ChPUM3/apum24*^*+/−*^ plants did not rescue that of *apum24*. Consistent with these complementation results, a known target RNA-binding sequence at the end of the 18S rRNA (5′-GGAAUUGACGG) for APUM23 was conserved in Arabidopsis and *C. corallina*, whereas a target region of ITS2 pre-rRNA for APUM24 was 156 nt longer in *C. corallina* than in *A. thaliana*. Moreover, ChPUM2 and APUM23 were predicted to have nearly identical structures, but ChPUM3 and APUM24 have different structures in the 5th C-terminal Puf RNA-binding domain, which had a longer random coil in ChPUM3 than in APUM24.

**Conclusions:**

ChPUM2 of *C. corallina* was functional in Arabidopsis, similar to APUM23, but ChPUM3 did not substitute for APUM24 in Arabidopsis. Protein homology modeling showed high coverage between APUM23 and ChPUM2, but displayed structural differences between APUM24 and ChPUM3. Together with the protein structure of ChPUM3 itself, a short ITS2 of Arabidopsis pre-rRNA may interrupt the binding of ChPUM3 to 3′-extended 5.8S pre-rRNA.

## Background

Pumilio proteins are a family of RNA-binding proteins that are evolutionarily conserved in eukaryotes [1]. Typical Pumilio proteins have tandem repeats of 8 Puf domains that recognize 8 RNA bases, and each Puf domain contains 35–39 amino acids that form three α-helical structures [2, 3]. The basis of RNA recognition by these proteins is the crescent-shaped structure [4, 5]. The conserved aromatic and basic amino acids on the concave side of the crescent structure interact with RNA, whereas the amino acids on the convex side interact with partner proteins. Although Pumilio proteins have a variety of biological roles, their major molecular functions are mRNA decay and localization, translational repression [6, 7], and rRNA processing [8]. Most of the Pumilio proteins are localized in the cytoplasm and are involved in the posttranscriptional regulation of mRNA. However, a small subset of these proteins is localized in the nucleolus and participates in rRNA processing. For instance, nucleolar Nop9 of yeast [9] and TbPUF7 of trypanosomes [8] are involved in 18S rRNA biosynthesis and ribosome maturation through proper pre-rRNA processing. In plants, two nucleolar Pumilio proteins have been implicated in rRNA processing [10–15], including Arabidopsis APUM23, a homolog of yeast Nop9, and APUM24, a homolog of human Puf-A and yeast Puf6. APUM23 not only is required for normal growth patterning, such as leaf development and organ polarity [10, 16] but also is involved in ABA signaling [17]. APUM24 is essential for plant development, as its homozygous mutant displays embryo lethality [15]. APUM24 is implicated in the maturation of 5.8S and 25S rRNAs, while APUM23 participates in the processing of 18S and 5.8S rRNAs.

Pre-rRNA is a long single-stranded RNA transcribed from the rDNA repeat in the nucleolus. This transcript is subsequently cleaved to three mature rRNAs (5.8S, 18S, and 25S) by endoribonucleolytic activities [18, 19]. Misprocessed rRNA byproducts that are produced during rRNA processing are degraded by 5′-to-3′ and 3′-to-5′ exoribonucleolytic activities. These two pre-rRNA processing activities require additional accessory proteins, such as RNA exosome components, Pumilio proteins, and many RNA-binding proteins. It has been reported that Arabidopsis and rice show similar pre-rRNA processing pathways, probably due to the similar flanking sequences around the endocleavage sites of A2 and A3 in ITS1 [20], suggesting that RNA binding specificity is essential for the selection of cleavage sites. Recently, APUM23 was found to bind 11 nt in the 18S rRNA at positions 1141–1151 [12], and APUM24 interacts with rRNA segments encompassing the 5.8S and ITS2 regions [15]. Therefore, it is likely that APUM23 and APUM24 play crucial roles in the recruitment of target RNA sequences and interacting proteins for the maturation of 18S and 5.8S rRNAs in Arabidopsis.

Approximately 450 million years ago, land plants evolved from Charophyta living in freshwater and adapted to the terrestrial environment [21–24]. Charophyta shares numerous molecular and physiological characteristics with living land plants that are not found in Chlorophyta [25, 26]. Charophyta shows high sequence similarities to land plants in plastidal *atpB* and *rbcL*, mitochondrial *nad5*, and nuclear-encoded small subunit rRNA genes [27].

In this study, we found that all the green plants (Viridiplantae) examined have two putative nucleolar Pumilio proteins homologous to Arabidopsis APUM23 and APUM24. Consistent with this, two nucleolar Pumilio genes, namely, *ChPUM2* and *ChPUM3*, were identified in *Chara corallina* by transcriptome analysis. We postulated that two Pumilio proteins encoded by these genes might be evolutionarily and functionally conserved, as their Arabidopsis homologs play crucial roles in pre-rRNA processing required for proper protein synthesis. Transiently expressed ChPUM2-RFP and ChPUM3-RFP were localized in the nucleoli of *Nicotiana benthamiana* leaf cells, suggesting the nucleolar function of ChPUM2 and ChPUM3. The *apum23* mutant transformed with *35S:ChPUM2* recovered its defective rRNA processing and morphological phenotypes to normal levels. However, the rRNA processing defects and embryo lethality of the *apum24* mutant were not rescued by *35S:ChPUM3* or *ChPUM2*. Consistent with the failure of complementation of *apum24* with *35S:ChPUM3*, APUM24 has different domain structures at the C-terminus from ChPUM3. Moreover, the target ITS2 region of Arabidopsis pre-rRNA is 156 nt shorter than that of *C. corallina* and might not be sufficient for the binding of ChPUM3.

## Results

### Phylogeny of nucleolar Pumilio proteins

Pumilio proteins are ubiquitous in eukaryotic organisms, albeit in different numbers [1, 5]. Among the organisms whose whole genome sequences are available, higher plants have a higher number of Pumilio proteins than photosynthetic single-cell organisms and nonplant organisms; for example, 25 Pumilio proteins are found in *Arabidopsis thaliana*, 20 in *Oryza sativa*, 14 in *Physcomitrella patens*, 5 in *Chlamydomonas reinhardtii*, 11 in *Caenorhabditis elegans*, 7 in *Saccharomyces cerevisiae*, and 2 in humans [10]. Based on a similarity search using Arabidopsis nucleolar Pumilio proteins (APUM23 and APUM24) as queries and the existence of a nucleolar localization signal(s) (NoLS) as a requirement [28], the green plants whose genomes have been sequenced (Phytozome v12.1; https://phytozome.jgi.doe.gov) were shown to have two putative nucleolar Pumilio proteins. Using PacBio Iso-Seq analysis, we also identified two putative nucleolar Pumilio proteins out of four Pumilio proteins in *C. corallina.* Consistent with our transcriptome analysis, four Pumilio proteins were predicted in the *Chara* genome data, including 2 nucleolar forms [26]. When compared with Arabidopsis APUMs, comprising 25 Pumilio proteins, ChPUM2 and ChPUM3 displayed high homology with APUM23 and APUM24, respectively, while ChPUM1 and ChPUM4 belonged to other distinct clades (Additional file 1: Figure S1).

**Fig. 1:**
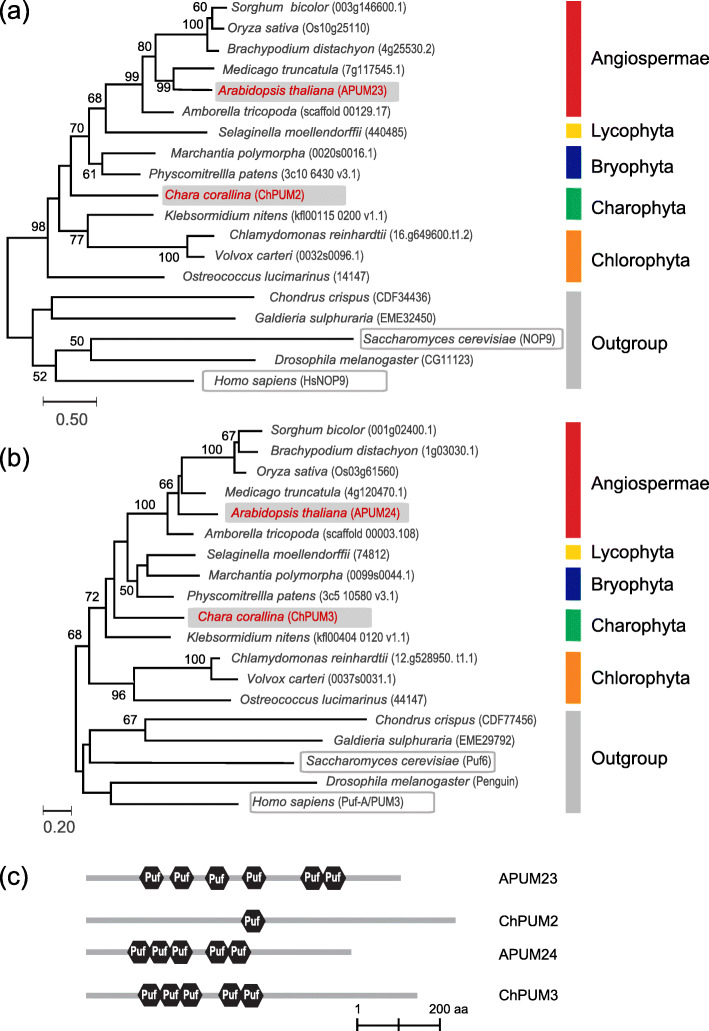
Phylogenetic trees of two nucleolar Pumilio protein families in the representative species of green plants (**a** and **b**) and the protein structures of nucleolar APUMs and ChPUMs (**c**). **a** and **b** Phylogenetic relationship among the putative nucleolar Pumilio proteins belonging to the APUM23 and ChPUM2 family (**a**) and the APUM24 and ChPUM3 family (**b**). The phylogenetic trees were constructed using the maximum likelihood LG + G model using MEGA7 software [29] with 1000 bootstrapping replicates. Two independent nucleolar Pumilio proteins of red algae (*Chondrus crispus* and *Galdieria sulphuraria*), *Drosophila melanogaster*, *Homo sapiens*, and *Saccharomyces cerevisiae* were used as outgroups. **c** Primary protein structures of APUM23 and APUM24 from *Arabidopsis thaliana* and ChPUM2 and ChPUM3 from *Chara corallina*. Black hexagons indicate Puf RNA-binding domains

To gain insight into the evolutionary relationship of putative nucleolar Pumilio proteins, we constructed a phylogenetic tree with the homologous proteins of 14 species of green plants together with 5 outgroup species that contain a NoLS(s) [28] (Fig. 1a and b). All green plants analyzed in this study had two proteins belonging to the APUM23 and APUM24 clades. The ChPUM2 of *C. corallina* was closer to APUM23 than APUM24, whereas ChPUM3 was categorized in the APUM24 clade. Phylogenetic analyses using ChPUM2 and ChPUM3 indicated that *C. corallina* is closer to land plants than other green algae examined in this study, suggesting that the evolution of nucleolar Pumilio proteins is consistent with previously determined phylogenetic positioning [21–25].

We then compared the number and position of Puf domains in ChPUM2, ChPUM3, APUM23, and APUM24 using the SMART web program (http://smart.embl-heidelberg.de) [30]. APUM23 and APUM24 have six and five Puf domains, respectively, and ChPUM2 and ChPUM3 have one and five Puf domains, respectively. As each Puf domain has been known to recognize a single RNA base [3], this observation raised the possibility that ChPUM2 may have a distinct RNA binding property from APUM23 and that ChPUM3 may bind similar, if not identical, RNA motifs as APUM24 (Fig. 1c).

### Structure of ChPUM2 and ChPUM3

**Fig. 2:**
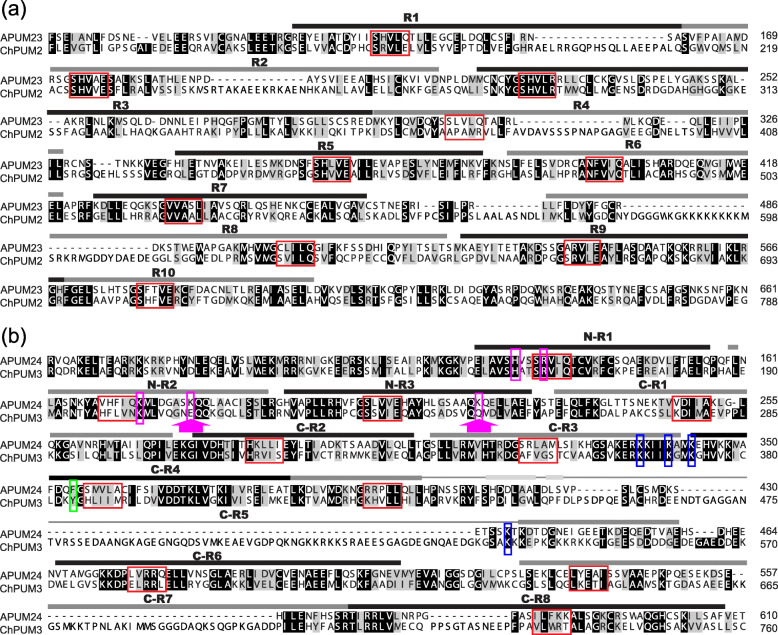
Amino acid sequence alignment of putative nucleolar Pumilio proteins, APUMs and ChPUMs. **a** Alignment of the amino acid sequences of APUM23 and ChPUM2. **b** Alignment of the amino acid sequences of APUM24 and ChPUM3. The Puf domains are indicated with black and gray lines above the amino acid sequences, and the five residues in the 2nd α-helix of each Puf domain that potentially interact with RNA bases are indicated with red boxes. The thin gray lines in the C-R5 Puf domain represent unfolded chains. Basic amino acids conserved in the N-terminal Puf domains of APUM24 family proteins are boxed in purple. Arrows under the purple box indicate the amino acids that are not conserved in ChPUM3. Conserved aromatic amino acids of APUM24 family proteins are boxed in green, and the basic amino acids in the C-terminal region are boxed in blue

Classic structural analysis of Pumilio proteins shows a tandem repeat of 8 Puf domains in the C-terminal region [31, 32]. However, a recent analysis of human Puf-A and yeast Puf6 identified 11 Puf domains, including 3 additional domains, in these Pumilio proteins involved in pre-rRNA processing [14]. A similar analysis previously performed for APUM23 showed 10 Puf domains instead of the six previously known domains [10, 12]. Consistent with a close phylogenetic relationship between APUM23 and ChPUM2 (Fig. 1a and Additional file 1: Figure S1), ChPUM2 also contained 10 Puf domains that showed an identical distribution as in APUM23 (Fig. 2a and Additional file 2: Figure S2a). Each domain of ChPUM2 showed an average 26% identity and 41% homology with the corresponding domain of APUM23. Notably, a high degree of homology was found in the 1st, 2nd, and 5th residues of the 2nd α-helix of each Puf domain (red boxes in Fig. 2a and Additional file 2: Figure S2a). These three residues have been known to play a pivotal role in the recognition of RNA bases [14].

Using the same approach, ChPUM3 was shown to possess 11 Puf domains (N-R1 to N-R3 at the N-terminus and C-R1 to C-R8 at the C-terminus) (Fig. 2b and Additional file 2: Figure S2b), as reported in human Puf-A and APUM24 [14]. Comparison of amino acids in 3 N-terminal Puf domains displayed an average identity of 47% and homology of 67% between APUM24 and ChPUM3, and that of 8 C-terminal domains showed 39% identity and 55% homology. Out of the 11 Puf domains, two domains (C-R5 and C-R7) had lower identities than the other domains (Fig. 2b).

**Fig. 3:**
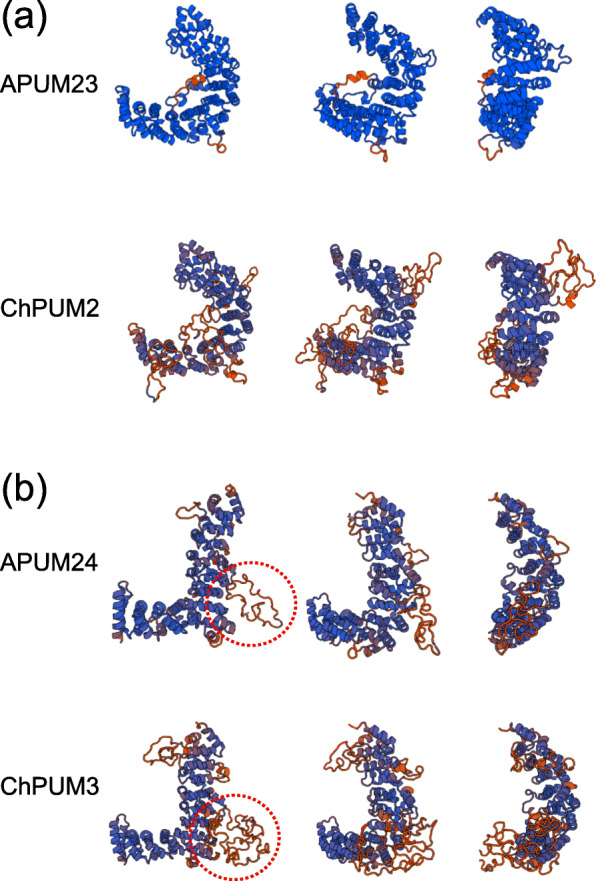
Predicted 3-D structures of putative nucleolar APUMs and ChPUMs. **a** C-shaped structures of APUM23 and ChPUM2. **b** L-shaped structures of APUM24 and ChPUM3. Unfolded side chains in the C-R5 domain are marked with red circles

Comparison of amino acid sequences among Puf domains suggested that ChPUM2 and ChPUM3 may be functional homologs of Arabidopsis APUM23 and APUM24, respectively (Fig. 2a and b), which is consistent with the observation obtained from phylogenetic analysis. However, since ChPUM2 and APUM23 contain different numbers of Puf domains, unlike ChPUM3 and APUM24, in the classic domain analysis (Fig. 1c), they may bind distinct RNA substrates. Therefore, to determine the structural relationship between nucleolar ChPUM and APUM proteins, we predicted the tertiary structure of these proteins using the SWISS-MODEL web server (https://swissmodel.expasy.org/) [33]. A previous high-resolution structural study demonstrated that the C-shaped structure of APUM23 has a long chain between the 2nd and 3rd α-helix of the R3 domain that participates in the recognition of RNA bases [12]. Homology modeling revealed a high similarity between ChPUM2 and the APUM23 reference protein, as well as the C-shaped structure similar to APUM23, in the 3-dimensional structure (Fig. 3a). Compared with APUM23, ChPUM2 has a long random coil in the R3 domain (red colored lines in the bottom panels of Fig. 3a), but it maintains uninterrupted 2nd and 3rd α-helical structures in this domain, similar to APUM23. Therefore, analyses of consensus amino acid sequences and homology modeling suggest that ChPUM2 may recognize similar, if not identical, RNA bases.

In contrast to the C-shaped configuration of ChPUM2 and APUM23, an L-shaped structure was predicted for ChPUM3 and APUM24, similar to the human Puf-A reference protein [14] (Fig. 3b). The most marked structural differences between ChPUM3 and APUM24 were found in the C-R5, and N-R2 and N-R3 domains. ChPUM3 had a longer random coil in the C-R5 domain than APUM24 (Fig. 2b and the dotted circles in Fig. 3b). Additionally, ChPUM3 contained negatively charged (E210) and uncharged (Q249) amino acids in the N-R2 and N-R3 domains (Fig. 2b), instead of the basic amino acids that are known to be involved in RNA binding and found at both sites of APUM24 and the human Puf-A reference [14]. Thus, it appeared that ChPUM3 has different RNA binding specificity from APUM24, considering the chain length of C-R5 and the lack of basic amino acids in 2 N-terminal Puf domains.

### Subcellular localization of ChPUM2 and ChPUM3

**Fig. 4:**
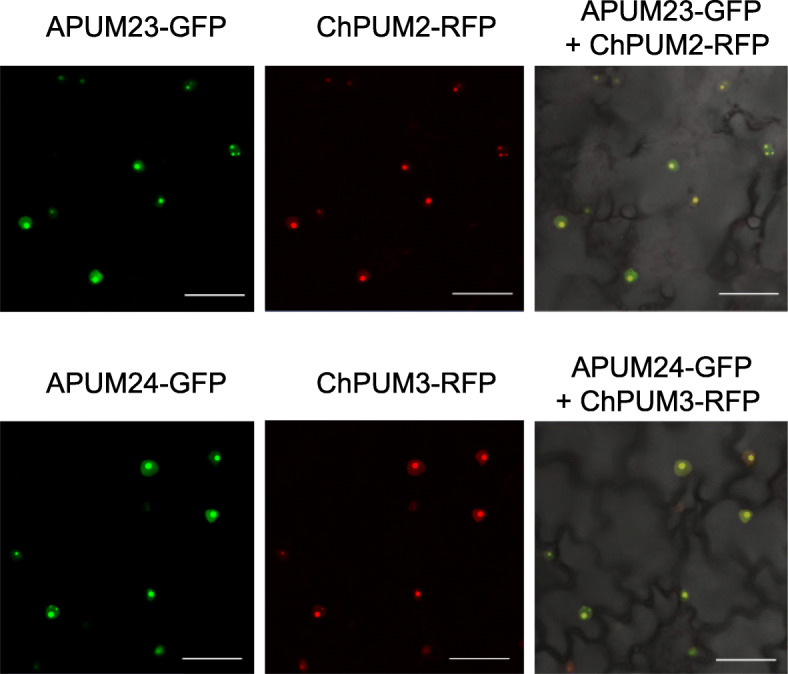
Nucleolar colocalization of APUM23-GFP and ChPUM2-RFP (upper panel) and APUM24-GFP and ChPUM3-RFP (lower panel) in *N. benthamiana* leaf cells. Scale bars = 50 μm

Next, we examined the subcellular localization of ChPUM2-RFP and ChPUM3-RFP. Previously, the GFP fusions of APUM23 and APUM24 were known to preferentially localize in the nucleoli of Arabidopsis root and tobacco leaf cells [10, 15]. We performed Agrobacterium-mediated coinfiltration into *N. benthamiana* leaf cells using *35S:APUM23-GFP* and *35S:ChPUM2-RFP* and *35S:APUM24-GFP* and *35S:ChPUM3-RFP*. All GFP- and RFP-tagged Pumilio proteins were found in the nucleoli and were weakly detected in the nucleoplasm (Fig. 4). The colocalization results suggest that ChPUM2 and ChPUM3 may play similar roles in pre-rRNA recognition and processing to APUM23 and APUM24.

### *ChPUM2* rescued the *apum23* mutant phenotype, but *ChPUM3* did not rescue *apum24*

**Fig. 5:**
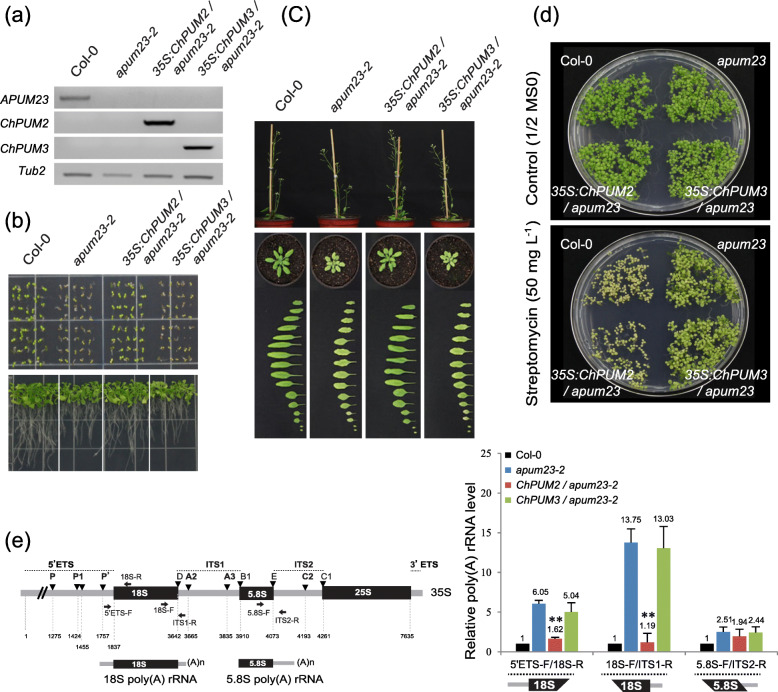
Complementation assays of *35S:ChPUM2* and *35S:ChPUM3* transgenic plants in the *apum23* mutant background. **a** Confirmation of the expression of *ChPUM2* and *ChPUM3* transgenes in the *apum23–2* mutant using RT-PCR. Gel images were processed from original figures (Additional file 3: Figure S3) by Photoshop CS5. **b** Normal germination and root growth of *apum23–2* complemented with *35S:ChPUM2.***c** Plant heights and rosette leaves in mature plants. Leaves were collected from 2-week-old plants. **d** Recovery of streptomycin susceptibility in the *apum23–2* complemented *35S:ChPUM2.***e** qRT-PCR analysis for unprocessed rRNAs in wild-type Col-0, *apum23–2*, *35S:ChPUM2/apum23–2*, and *35S:ChPUM3/apum23–2*. Two technical and three biological replicates were performed for PCR measurements. Asterisks indicate the results of Student’s *t*-test between *apum23–2* and transgenic plants (**; *p < 0.01*). Values represent means ± standard deviations, SDs (*n* = 3). The left panel shows a schematic diagram of poly(A) pre-rRNA byproducts and the primers used for the detection of pre-rRNAs. Unprocessed poly(A) 18S (~ 2.6 knt) and 5.8S (~ 300 nt) pre-rRNAs are shown below the 35S pre-rRNA

**Fig. 6:**
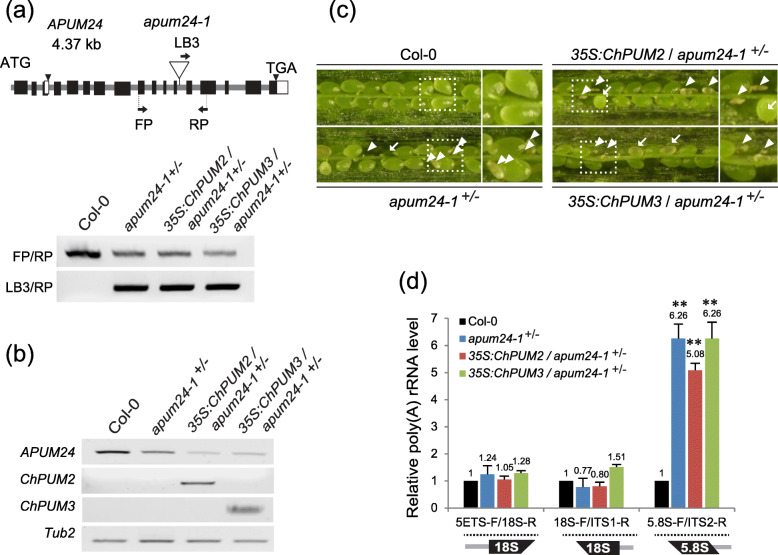
Complementation assays of *35S:ChPUM2* and *35S:ChPUM3* transgenic plants in the *apum24*^*+/−*^ mutant background. **a** T-DNA insertion site of *apum24–1* mutant alleles (upper panel) and genotyping (bottom panel). Primers used for genotyping are indicated with arrows. Original gel images for bottom panel are provided in Additional file 4: Figure S4a. **b** Confirmation of the expression of *ChPUM2* and *ChPUM3* transgenes in the *apum24–1*^*+/−*^ mutant using RT-PCR. Original gel images are provided in Additional file 4: Figure S4b. **c** Siliques of Col-0 control, *apum24–1*^*+/−*^, and transgenic *apum24–1*^*+/−*^ expressing *35S:ChPUM2* or *35S:ChPUM3*. The right panels for each plant line are enlarged images of the boxed regions. Arrows and arrowheads indicate undeveloped ovules and aborted seeds, respectively. Note that none of the transgenics complemented the abnormal seeds to normal levels. **d** qRT-PCR for analyzing relative unprocessed rRNA levels in Col-0 control, *apum24–1*^*+/−*^, and *35:ChPUMN*/*apum24–1*^*+/−*^ using the same primers that were used in Fig. 5. Two technical and three biological replicates were performed for PCR measurements. Values represent means ± SDs (*n* = 3) (**; *p < 0.01)*

To assess whether ChPUM2 and ChPUM3 are involved in nucleolar functions similar to APUM23 and APUM24, complementation assays were performed on homozygous *apum23* (Fig. 5) and heterozygous *apum24* (Fig. 6) mutants by transforming the *35S:ChPUM2* and *35S:ChPUM3* constructs*.* While the *apum23–2* mutant showed the phenotypes of delayed germination (Fig. 5a and b), short roots (bottom panel in Fig. 5b), short inflorescence stems (upper panel in Fig. 5c), light green leaves (bottom panel in Fig. 5c), and streptomycin resistance (Fig. 5d), the *35S:ChPUM2/apum23–2*^*−/−*^ plants exhibited normal phenotypes (Fig. 5a-d). Notably, the recovered streptomycin susceptibility of *35S:ChPUM2/apum23–2*^*−/−*^ plants indicates the normal ribosomal functions of complemented plants (Fig. 5d). In contrast to *35S:ChPUM2/apum23–2*^*−/−*^ plants, *35S:ChPUM3/apum23–2*^*−/−*^ plants maintained an *apum23–2* mutant phenotype (Fig. 5a-d). The failure to restore the *apum23–2* phenotype with *35S:ChPUM3* excluded the possibility that ChPUM3 and APUM23 are orthologous proteins.

In addition to the restoration of morphological phenotypes, *35S:ChPUM2*/*apum23–2*^*−/−*^ rescued the defects observed in the *apum23–2*^*−/−*^ mutant that accumulates poly (A)-tailed 5′ETS-18S-ITS1 and 5.8S-ITS2 pre-rRNAs (Fig. 5e). The poly (A) pre-rRNAs were detected using quantitative reverse transcriptase-PCR (qRT-PCR) and three combinations of primers. In the *apum23–2*^*−/−*^ mutant, all three qRT-PCR products (5′ETS-18S, 18S-ITS1, and 5.8S-ITS2) were accumulated, but in *35S:ChPUM2*/*apum23–2*^*−/−*^ plants, the amounts of poly(A)-tailed 18S-ITS1 and 5.8S-ITS2 pre-rRNAs were greatly reduced compared with those in *apum23–2*^*−/−*^*,* although the amount of poly(A) 5.8S-ITS2 was slightly decreased. In contrast to *35S:ChPUM2*/*apum23–2*^*−/−*^, *35S:ChPUM3*/*apum23–2*^*−/−*^ showed a nearly identical amount of poly(A) pre-rRNAs to the *apum23–2*^*−/−*^ mutant (Fig. 5e, right panel). The qRT-PCR results indicate that ChPUM2 is involved in a similar pre-rRNA processing pathway as APUM23, although it was not fully functional in the removal of the 5.8S pre-rRNA byproducts.

In contrast to the restoration of *apum23* by the *ChPUM2* transgene, the *apum24* phenotype was not restored by the *ChPUM2* or *ChPUM3* transgenes. As the homozygous *apum24*^*−/−*^ mutant is lethal [13, 15, 34], the heterozygous *apum24–1*^*+/−*^ mutant was used for complementation analysis. The *35S:ChPUM3/apum24–1*^*+/−*^ plants set normal and abnormal seeds at similar rates as the *apum24*^*+/−*^ mutant (Fig. 6a-c and Table 1), showing 30.7 and 33.2% abnormal seeds for the *35S:ChPUM3/apum24–1*^*+/−*^ and *apum24–1*^*+/−*^ plants, respectively. As expected, the *35S:ChPUM2/apum24–1*^*+/−*^ plants produced abnormal seeds (32.5%) at a similar ratio to the *35S:ChPUM3/apum24–1*^*+/−*^ plants. Consistent with this result for the morphological phenotype, *35S:ChPUM2*/*apum24–1*^*+/−*^ and *35S:ChPUM3*/*apum24–1*^*+/−*^ plants accumulated poly(A)-tailed 18S-ITS1 and 5.8S-ITS2 pre-rRNA similar to the *apum24–1*^*+/−*^ plants (Fig. 6d), indicating that ChPUM3 is not a functional ortholog of Arabidopsis APUM24.

**Table 1:** Ratio of normal to abnormal seeds

Genotype	Number of			Ratio (N:A)
	total seeds	normal seeds (N)	abnormal seeds (A)^a^	
Control (pB2GW7)	3016	2869	147	19.5 : 1
*apum24–1*^*+/−*^	3043	2108	935	2.25: 1
*35S:ChPUM2* / *apum24–1*^*+/−*^	3035	2050	985	2.08: 1
*35S:ChPUM3* / *apum24–1*^*+/−*^	2988	1997	991	2.01: 1

^a^aborted seeds and undeveloped ovules

### *ChPUM2* restored the salt- and glucose-hypersensitive phenotypes of *apum23*, but *ChPUM3* did not restore the *apum23* and *apum24* phenotypes

**Fig. 7:**
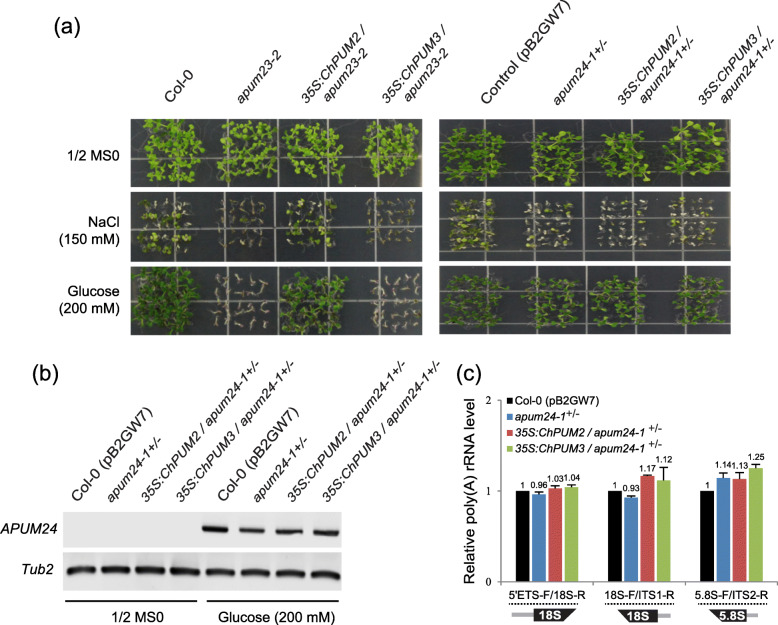
Restoration analyses of the salt- and sugar-sensitive *apum23* and *apum24* phenotypes by *ChPUM2* and *ChPUM3*. **a***apum23–2* (left panel) and *apum24–1*^*+/−*^ (right panel) seedlings expressing either *35S:ChPUM2* or *35S:ChPUM3* in the absence or presence of NaCl and glucose. Col-0 Control (pB2GW7), *apum24–1*^*+/−*^, *35S:ChPUM2/apum24–1*^*+/−*^, and *35S:ChPUM3/apum24–1*^*+/−*^ seeds were germinated on 1/2 MS medium containing 10 mg L^− 1^ Basta with the indicated treatment. **b** Expression levels of *APUM24* in Col-0 Control (pB2GW7), *apum24–1*^*+/−*^, and transgenic *apum24–1*^*+/−*^ plants expressing *35S:ChPUM2* and *35S:ChPUM3* in the absence and presence of 200 mM glucose. Note the similar expression levels of *APUM24* in the presence of 200 mM glucose. Original gel images are provided in Additional file 5: Figure S5. **c** qRT-PCR analysis of the relative unprocessed rRNA levels in Col-0 Control (pB2GW7), *apum24–1*^*+/−*^, and transgenic *apum24–1*^*+/−*^ plants expressing *35S:ChPUM2* and *35S:ChPUM3* in the presence of 200 mM glucose. The same primers were used as in Fig. 5. Two technical and three biological replicates were performed for PCR measurements. Values represent means ± SDs (*n* = 3).

*apum23* and *apum24* showed changes in the expression levels of ribosomal biogenesis-related genes, which in turn resulted in hypersensitivity to high concentrations of salt in *apum23* [17] and glucose in a weak *apum24* mutant [13]. We therefore examined whether *ChPUM2* and *ChPUM3* could restore the altered physiological phenotypes of *apum23*^*−/−*^ and *apum24*^*+/−*^. The *35S:ChPUM2/apum23–2*^*−/−*^ seedlings exhibited a similar degree of resistance to 150 mM NaCl and 200 mM glucose as wild-type Col-0 seedlings, while the *35S:ChPUM3/apum23–*2^*−/−*^ seedlings showed NaCl and glucose susceptibility similar to that of *apum23–2*^*−/−*^ seedlings (left panel in Fig. 7a). However, *35S:ChPUM2/apum24–1*^*+/−*^ and *35S:ChPUM3/apum24–1*^*+/−*^ failed to recover the salt sensitivity of *apum24–1*^*+/−*^ (right panel in Fig. 7a). Unexpectedly, when *35S:ChPUM2* or *35S:ChPUM3* was overexpressed in *apum24–1*^*+/−*^, their transgenic seedlings showed a similar glucose resistance as that found in wild-type and *apum24–1*^*+/−*^ seedlings (right panel in Fig. 7a). It was previously reported that *APUM24* gene expression was greatly increased in wild-type plants by exogenously supplied glucose [13, 15]. Our data showed similar expression levels of the *APUM24* transcript in the Col-0 (pB2GW7) control, *apum24–1*^*+/−*^, *35S:ChPUM2/apum24–1*^*+/−*^, and *35S:ChPUM3/apum24–1*^*+/−*^ in the presence of 200 mM glucose (Fig. 7b). The normal growth of *apum24–1*^*+/−*^ suggests that heterozygotic expression of *APUM24* might be sufficient for the glucose-induced phenotype. Similar to *apum24–1*^*+/−*^, the *apum24–1*^*+/−*^ plants transformed with *35S:ChPUM2* or *35S:ChPUM3* grew normally under glucose treatment, probably owing to the expression of heterozygotic *APUM24*. Moreover, all the plants showed similar amounts of unprocessed 5.8S rRNAs as Col-0 (pB2GW7) control under 200 mM glucose treatment (Fig. 7c), while they displayed 5.08- to 6.26-fold higher amounts of unprocessed 5.8S rRNAs than the Col-0 (pB2GW7) control (Fig. 6d). This result suggests that glucose supplementation of heterozygous *apum24*^*+/−*^ increased the level of heterozygous *APUM24* up to the homozygous *APUM24* level. Therefore, the normal phenotype and 5.8S pre-rRNA processing that were observed in *35S:ChPUM2/apum24–1*^*+/−*^ and *35S:ChPUM3/apum24–1*^*+/−*^ plants resulted from increased levels of Arabidopsis APUM24 caused by exogenously supplied glucose.

## Discussion

Based on our transcriptome data, public databases, and previous results [10, 15, 35], we found that the green plants examined in this study have two nucleolar Pumilio proteins. Protein phylogeny showed a closer relationship of nucleolar Pumilio proteins of land plants with multicellular *C. corallina* than with single-celled green algae (Chlorophyta). This result is consistent with a previous report showing that the genus *Chara* is more closely related to land plants than other green plants [27]. Although the possibility that certain putative nucleolar Pumilio proteins do not have a nucleolar function has not been ruled out, land plant species appear to have evolved two Pumilio proteins for the removal of aberrant pre-rRNAs.

We demonstrated that ChPUM2 is a functional ortholog of APUM23 in Arabidopsis cells, as evident from the restoration of defective pre-rRNA processing and morphology of *apum23* in *35S:ChPUM2/apum23* plants. Arabidopsis nucleolar Pumilio proteins are known to play a role in recognizing the target sequences on pre-rRNA and recruiting catalytic proteins such as exoribonuclease [10, 12, 13, 15]. Comparison of pre-rRNA identified a target sequence (5′-GGAAUUGACGG-3′) of APUM23 [12] in the 18S rRNA of *C. corallina* at positions 1148–1158 (Additional file 6: Figure S6). Therefore, it is likely that ChPUM2 may bind to this sequence in *C. corallina*. This assumption is supported by the primary structure of ChPUM2 and APUM23, each Puf domain of which is highly conserved between ChPUM2 and APUM23 except the fourth domain (Fig. 2a). Therefore, the common target rRNA sequence and similar amino acid composition of Puf domains might enable the restoration of *apum23* phenotypes, including morphological and rRNA processing defects, to normal in *35S:ChPUM2/apum23*. In *apum23* complementation analysis using *35S:ChPUM2*, the poly(A)-tailed 5.8S pre-rRNAs that accumulated in the *apum23* mutant were not completely removed (Fig. 5e). It seems possible that this might be due to a weak interaction of ChPUM2 with other unknown proteins that belong to the partners of intrinsic APUM23 specifically required for 5.8S rRNA processing. Indeed, the predicted structure of ChPUM2 has more unfolded chains than APUM23 (Fig. 3a), which may interfere with the interaction of ChPUM2 with other protein components. To verify this possibility *in planta*, it is worthwhile to identify and compare the components interacting with CPUM2 in *C. corallina* and APUM23 in Arabidopsis.

Although ChPUM3 appeared structurally similar to APUM24 (Fig. 3b), ChPUM3 did not functionally replace APUM24 in Arabidopsis. We assume that this result is due to fine structural differences between ChPUM3 and APUM24. Typical Pumilio proteins bind to a specific RNA base with the second α-helix of the Puf domain, but APUM24 and its homologs are not capable of binding to a specific RNA base through this α-helix domain [14, 15]. ChPUM3 does not complement the *apum24* mutant perhaps due to (1) the very long random coil in the C-R5 domain, (2) the negatively charged and uncharged amino acids in two N-terminal domains, and (3) the long ITS2 sequence in *C. corallina*. First, a very long random chain at the C-R5 domain of ChPUM3 would interrupt the interaction of other C-terminal domains with RNA bases in the 5.8S-ITS2 junction of Arabidopsis pre-rRNA. Indeed, it was reported that human Puf-A and its homolog APUM24 have a long random coil in C-R5 that prevents the C-terminal Puf domains from binding to RNA [14]. ChPUM3 has a random coil that is an 80 aa longer than that of APUM24 (Fig. 2b); thus, ChPUM3 may not recognize Arabidopsis pre-rRNA. Second, in ChPUM3, the N-R2 and N-R3 of patch 1B include negatively charged (E210) and uncharged (Q249) amino acids, unlike the positive amino acids (K) at both positions of APUM24, which may result in differential binding characteristics from APUM24 toward the 5.8S-ITS2 region. The N-R2 and N-R3 domains of the human Puf-A protein are essential for RNA binding [14]. Third, ChPUM3 might be optimized for the recognition of long ITS2 sequences. ITS2 of *C. corallina* pre-rRNA is 156 nt longer than that of Arabidopsis (Additional file 7: Figure S7). In addition to the long side chain of the C-R5 domain in ChPUM3, the relatively short ITS2 sequence of Arabidopsis pre-rRNA may prevent ChPUM3 from binding to its substrate. Indeed, ITS2 evolved rapidly and has been used to evaluate genetic divergence [36, 37].

## Conclusions

In this study, we identified two nucleolar Pumilio proteins, namely, ChPUM2 and ChPUM3, from *C. corallina* that are phylogenetically and structurally close to the Arabidopsis nucleolar Pumilio proteins APUM23 and APUM24, respectively. Complementation analyses using *35S:ChPUM2* and *35S:ChPUM3* showed that *ChPUM2* rescued the defective phenotypes of the *apum23* mutant, but *ChPUM3* did not restore the phenotypes of the *apum24* mutant. Consistent with these complementation results, ChPUM2 showed similar features of Puf domains as APUM23 in the primary amino acid sequence and a predicted 3-D protein structure. ChPUM3 has a long random coil in the C-R5 domain and contains distinct amino acids from those in APUM24 in the N-terminal domain. In addition to the structural difference between ChPUM3 and APUM24, a short ITS2 sequence of Arabidopsis pre-rRNA might prevent ChPUM3 from properly processing Arabidopsis 5.8S pre-rRNA. Taken together, the results show that ChPUM2 was functional in Arabidopsis, similar to APUM23, but ChPUM3 could not substitute for APUM24 in Arabidopsis. Further studies on the nucleolar functions of ChPUM2 and ChPUM3 in Charophyta will help us understand the evolution of rRNA processing in green plants.

## Methods

### Plant materials and growth conditions

*C. corallina* was collected at the private land (38°33′N, 128°50′E) in South Korea with the kind permission of land owner, and grown at room temperature in a small aquarium. Genomic DNA and voucher specimens of *C. corallina* were formally identified by Dr. Min Ha Kim at the Plant Resources Division of the National Institute of Biological Resources (https://www.nibr.go.kr/) and deposited under the number NIBRGR0000609814. The *apum23* [10] and *apum24* [15] mutants, obtained from Arabidopsis Biological Resources Center and reported previously, were used for complementation analyses. The *35S:ChPUM2* and *35S:ChPUM3* transgenic Arabidopsis plants in the *apum23* and *apum24* backgrounds were produced by transformation using *Agrobacterium tumefaciens* GV3101 with the floral dipping method [38]. *A. thaliana* wild-type Col-0 and control (Col-0 transformed with pB2GW7) and the *apum23–2*^*−/−*^*, apum24–1*^*+/−*^, *35S:ChPUM2*, and *35S:ChPUM3* overexpression lines were grown on MS medium or in the soil at 22 °C under 16 h light (120 μmol photons m^− 2^ s^− 2^) and 8 h dark cycles. For testing antibiotic, salt, and glucose resistance, seeds were germinated on 1/2 MS plates supplemented with 50 mg L^− 1^ streptomycin, 150 mM NaCl, and 200 mM glucose, respectively, in a growth room for 10 to 12 days. All seeds were stratified at 4 °C for 3 days before sowing.

### Identification of *ChPUM2* and *ChPUM3* transcripts

The cDNA sequences of Pumilio proteins of *C. corallina* (*ChPUM*s) were obtained by searching our PacBio Iso-Seq transcriptome data that were generated from whole plants, including thallus, rhizoids, globules (antheridia), and nucules (archegonia). The expression of *ChPUM*s was verified using RT-PCR. Nucleolar ChPUMs were identified by the comparison of Arabidopsis nucleolar Pumilio proteins (APUM23 and APUM24) with Pumilio proteins of *C. corallina* and shown to have a NoLS [28].

### Phylogenic analysis of ChPUM2 and ChPUM3 homologs

To perform the phylogenetic analysis, COGs (Clusters of Orthologous Groups) of amino acid sequences of the proteins of ChPUM2, ChPUM3, APUM23, and APUM24 were obtained from representative species of Viridiplantae (green plants) in Phytozome (v 12.1) [35], *Klebsormidium flaccidum* in the Klebsormidium genome database (http://www.plantmorphogenesis.bio.titech.ac.jp/~algae_genome_project/klebsormidium/), and two red algae species in the Ensembl Plant database (http://plants.ensembl.org). A phylogenetic tree was constructed by using the maximum likelihood method based on the LG + G model with MEGA7 software [29, 39]. Amino acid sequence alignments were performed using ClustalW (http://www.clustal.org/) and edited using BioEdit software (https://bioedit.software.informer.com/).

### Plasmid construction

For the construction of *35S:ChPUM* plasmids, coding sequences (CDSs) for *ChPUM2* and *ChPUM3* were amplified by RT-PCR using the primers ChPUM2-F and ChPUM2-R1 and ChPUM3-F and ChPUM3-R1, respectively (see Additional file 8: Table S1 for primer sequences). The PCR products were inserted into the pENTR-D-TOPO vector (Invitrogen) and then transferred to pB2GW7 or pK2GW7 (Vlaams Instituut voor Biotechnologie, Ghent University) by Gateway™ LR Cloase II (Invitrogen). For the construction of *35S:ChPUM-RFP* and *35S:APUM-GFP* plasmids, CDSs of *ChPUM2*, *ChPUM3*, *APUM*23, and *APUM24* were amplified by RT-PCR using the primer combinations ChPUM2-F/ChPUM2-R2, ChPUM3-F/ChPUM3-R2, APUM23-F/APUM23-R, and APUM24-F/APUM24-R, respectively. PCR products were inserted into the pENTR-D-TOPO vector and then transferred to the pB7RWG2 or pK7FWG2 vector [40] by Gateway™ LR Clonase II.

### Colocalization assay of ChPUM and APUM fusion proteins

The C-terminal RFP fusion proteins of ChPUM2 and ChPUM3 and C-terminal GFP fusions of APUM23 and APUM24 were transiently expressed in *N. benthamiana* leaves using agroinfiltration [41]. Briefly, cultures of Agrobacterium carrying fusion constructs were harvested at the stationary phase and resuspended in MMA buffer (10 mM MES, 10 mM MgCl_2_, and 150 μM acetosyringone) to OD_600_ = 0.8. For coexpression of ChPUM-RFP and APUM-GFP, equal volumes of two Agrobacterium cultures that had either the *35S:ChPUM-RFP* or *35S:APUM-GFP* construct were mixed before infiltration. Infiltration was performed on the abaxial side of tobacco leaves using a needleless syringe. Plants were kept in the dark at 22 °C under high humidity for 30–34 h, and the infiltrated leaves were observed under a fluorescence microscope.

### Quantitative reverse transcriptase-PCR (qRT-PCR) for analyzing unprocessed rRNA

Total RNA was isolated using the RNeasy Plant Mini Kit (Qiagen, cat. # 74904) from 100 mg seedling and treated with 2 units of RNase-free TURBO™ DNase (Ambion, cat. # AM2238) in 50 μL reaction at 37 °C for 50 min. First-strand cDNA was synthesized from 5 μg of total RNA using the oligo (dT)_18_ primer in a 20 μL reaction and diluted 3-fold. Then, one μL of cDNA was mixed with 0.6 μL of 10 mM primers and 10 μL of 2 x SYBR® Green Supermix (Bio-Rad, cat. # 172–5261) in a 20 μL reaction and subjected to PCR according to the manufacturer’s instructions. For the detection of unprocessed poly(A) rRNAs, three different combinations of primers (5′ETS/18S, 18S/ITS1, and 5.8S/ITS2) were used. Tubulin (*Tub4,* At5g44340) cDNA was used as an internal control. For qPCR measurements, two technical and three biological replicates were used. Data were calculated using the 2^-ΔΔCT^ method [42].

## Supplementary information

**Additional file 1: Figure S1.** Phylogenetic tree of the Pumilio proteins using 25 APUMs from *A. thaliana* APUMs and 4 ChPUMs from *C. corallina*. The maximum likelihood tree was generated using the JTT + F + G model with 1000 bootstrapping replicates.

**Additional file 2: Figure S2.** Alignment of five RNA recognition residues in each Pumilio repeat from nucleolar APUMs and ChPUMs. Five residues of each Pumilio repeat in APUM23 and APUM24 known as classically important for RNA recognition are aligned with those of their homologous ChPUMs, namely, ChPUM2 and ChPUM3, respectively.

**Additional file 3: Figure S3.** Original agarose gel images of RT-PCR products for Fig. 5a.

**Additional file 4: Figure S4.** Original agarose gel images of RT-PCR products for Fig. 6a and b.

**Additional file 5: Figure S5.** Original agarose gel images of RT-PCR products for Fig. 7b.

**Additional file 6: Figure S6.** Alignment of 18S rRNA sequences of *A. thaliana* and *C. corallina*. The red box indicates the Arabidopsis rRNA sequence at nt positions 1141–1151 to which APUM23 binds, and the blue box shows the identical sequence at nt positions 1148–1158 of 18S rRNA in *C. corallina*.

**Additional file 7: Figure S7.** Alignment of 5.8S rRNA and ITS2 sequences of *A. thaliana* and *C. corallina****.*** (a) Alignment of 5.8S rRNA sequences. (b) Alignment of ITS2 sequences.

**Additional file 8: Table S1.** Primers used in this study.

## Data Availability

Sequence data for the cDNAs of *ChPUM2* and *ChPUM3* described in this study can be found in the GenBank database (https://www.ncbi.nlm.nih.gov/) under the accession numbers MN652915 and MN652916, respectively. The transcriptome dataset analyzed during the identification of *ChPUM2* and *ChPUM3* transcripts is available in the GenBank database (NCBI SRA accession number; SRP249259).

## Abbreviations

C-R: C-terminal repeat
ITS: Internal transcribed sequence
MS: Murashige and Skoog
N-R: N-terminal repeat
pre-rRNA: pre-ribosomal RNA
