# Supplementary material for: Structural and functional similarities and differences in nucleolar Pumilio RNA-binding proteins between Arabidopsis and the charophyte Chara corallina
Source: BMC Plant Biol. 2020 May 24;20:230. doi: 10.1186/s12870-020-02444-x (PMC7247198; doi:10.1186/s12870-020-02444-x)
Supplement: Supplementary file 1 — Additional file 1: Figure S1. Phylogenetic tree of the Pumilio proteins using 25 APUMs from A. thaliana APUMs and 4 ChPUMs from C. corallina. The maximum likelihood tree was generated using the JTT + F + G model with 1000 bootstrapping replicates. [file 12870_2020_2444_MOESM1_ESM.pdf]

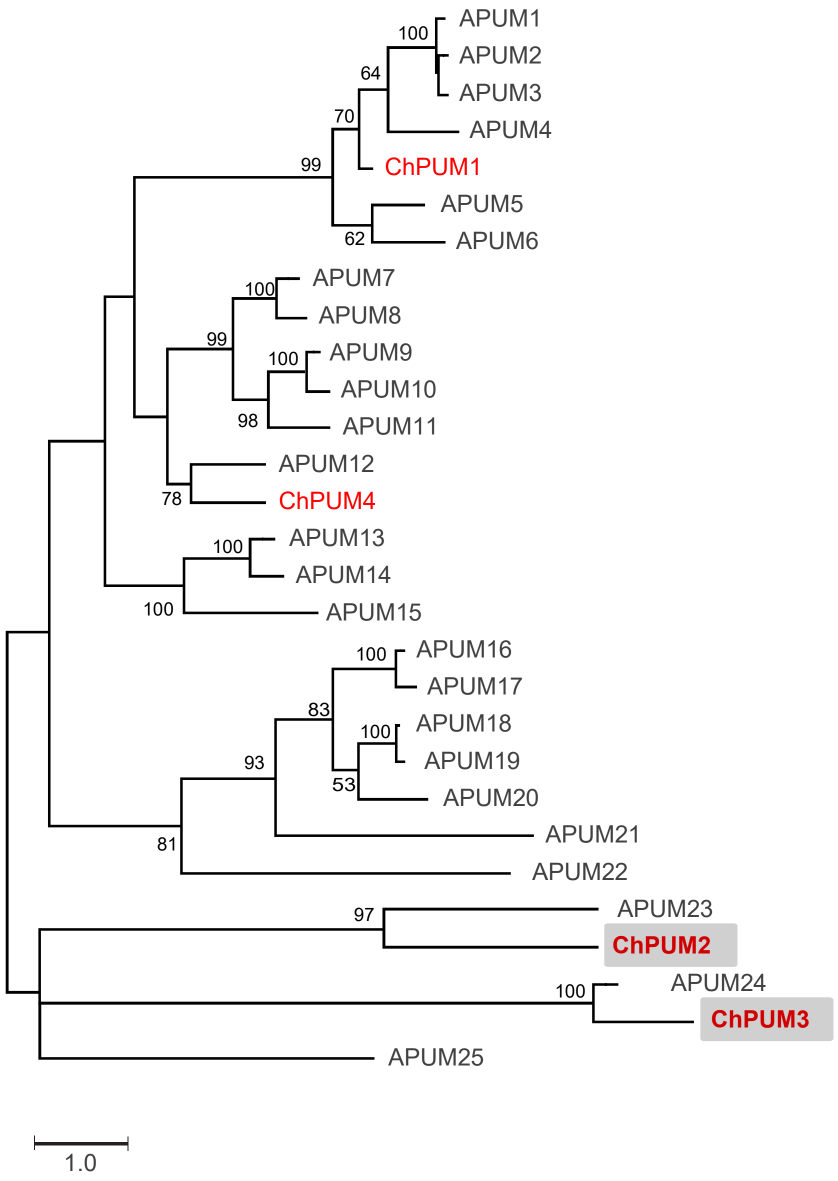

Figure S1. Phylogenetic tree of the Pumilio proteins using 25 APUMs from *A. thaliana* APUMs and 4 ChPUMs from *C. corallina*. The maximum likelihood tree was generated using the JTT+F+G model with 1000 bootstrapping replicates.
