# Supplementary material for: Structural and functional similarities and differences in nucleolar Pumilio RNA-binding proteins between Arabidopsis and the charophyte Chara corallina
Source: BMC Plant Biol. 2020 May 24;20:230. doi: 10.1186/s12870-020-02444-x (PMC7247198; doi:10.1186/s12870-020-02444-x)
Supplement: Supplementary file 2 — Additional file 2: Figure S2. Alignment of five RNA recognition residues in each Pumilio repeat from nucleolar APUMs and ChPUMs. Five residues of each Pumilio repeat in APUM23 and APUM24 known as classically important for RNA recognition are aligned with those of their homologous ChPUMs, namely, ChPUM2 and ChPUM3, respectively. [file 12870_2020_2444_MOESM2_ESM.pdf]

(a)

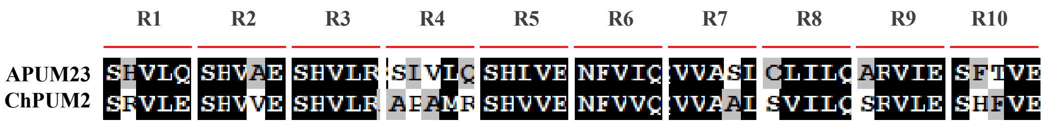

(b)

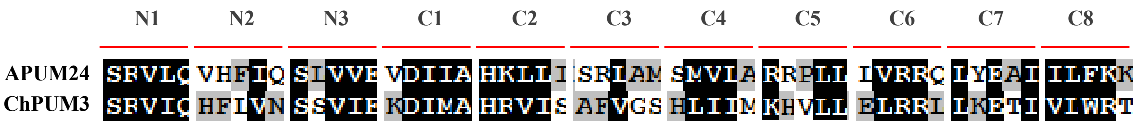

Figure S2. Alignment of five RNA recognition residues in each Pumilio repeat from nucleolar APUMs and ChPUMs. Five residues of each Pumilio repeat in APUM23 and APUM24 known as classically important for RNA recognition are aligned with those of their homologous ChPUMs, namely, ChPUM2 and ChPUM3, respectively.
