# Supplementary figures and images for: Structural and functional similarities and differences in nucleolar Pumilio RNA-binding proteins between Arabidopsis and the charophyte Chara corallina
Source: BMC Plant Biol. 2020 May 24;20:230. doi: 10.1186/s12870-020-02444-x (PMC7247198; doi:10.1186/s12870-020-02444-x)

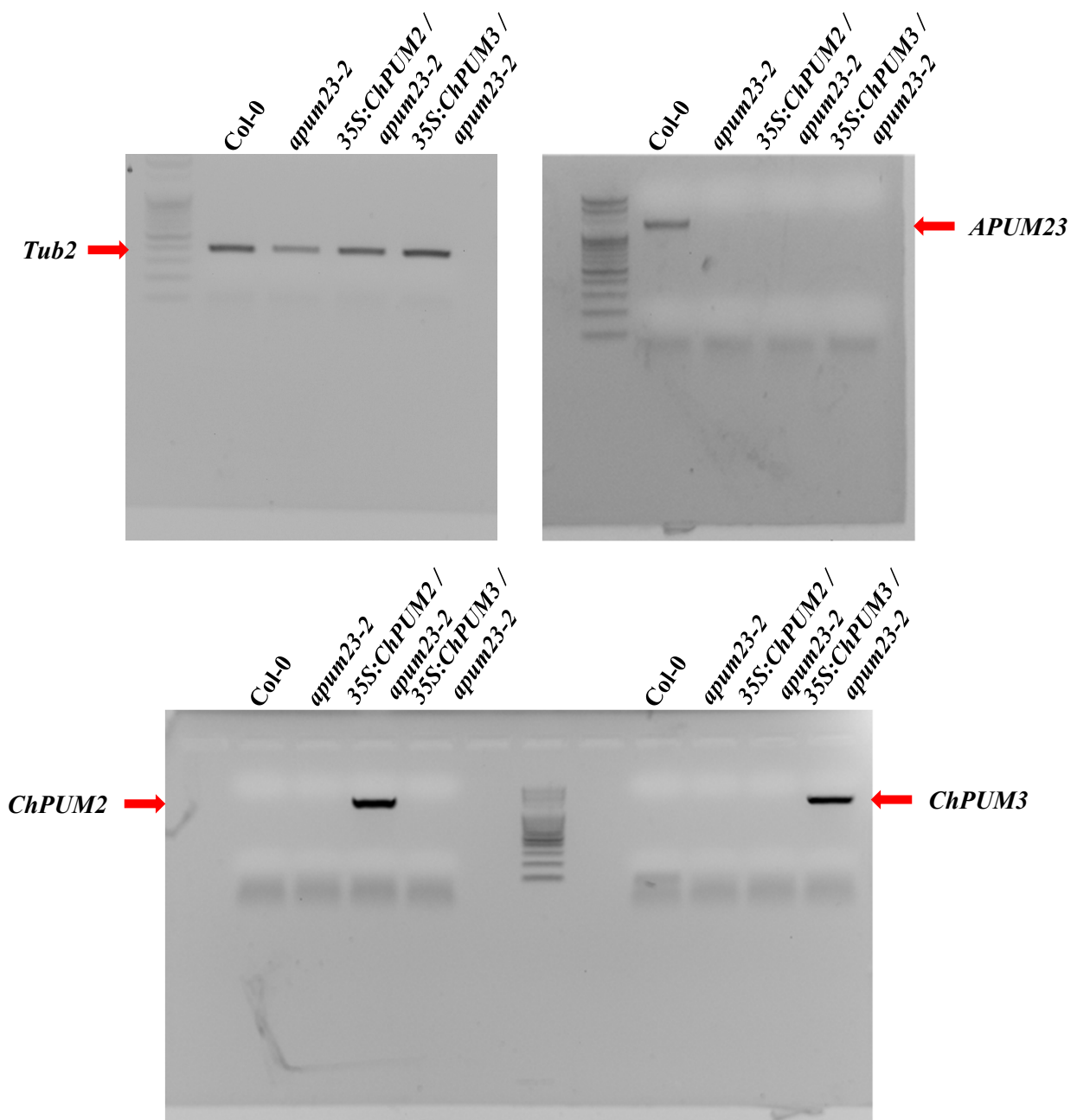

**Figure S3.** Original agarose gel images of RT-PCR products for Fig. 5a.

Supplement: Supplementary file 3 — Additional file 3: Figure S3. Original agarose gel images of RT-PCR products for Fig. 5a. [file 12870_2020_2444_MOESM3_ESM.pdf]

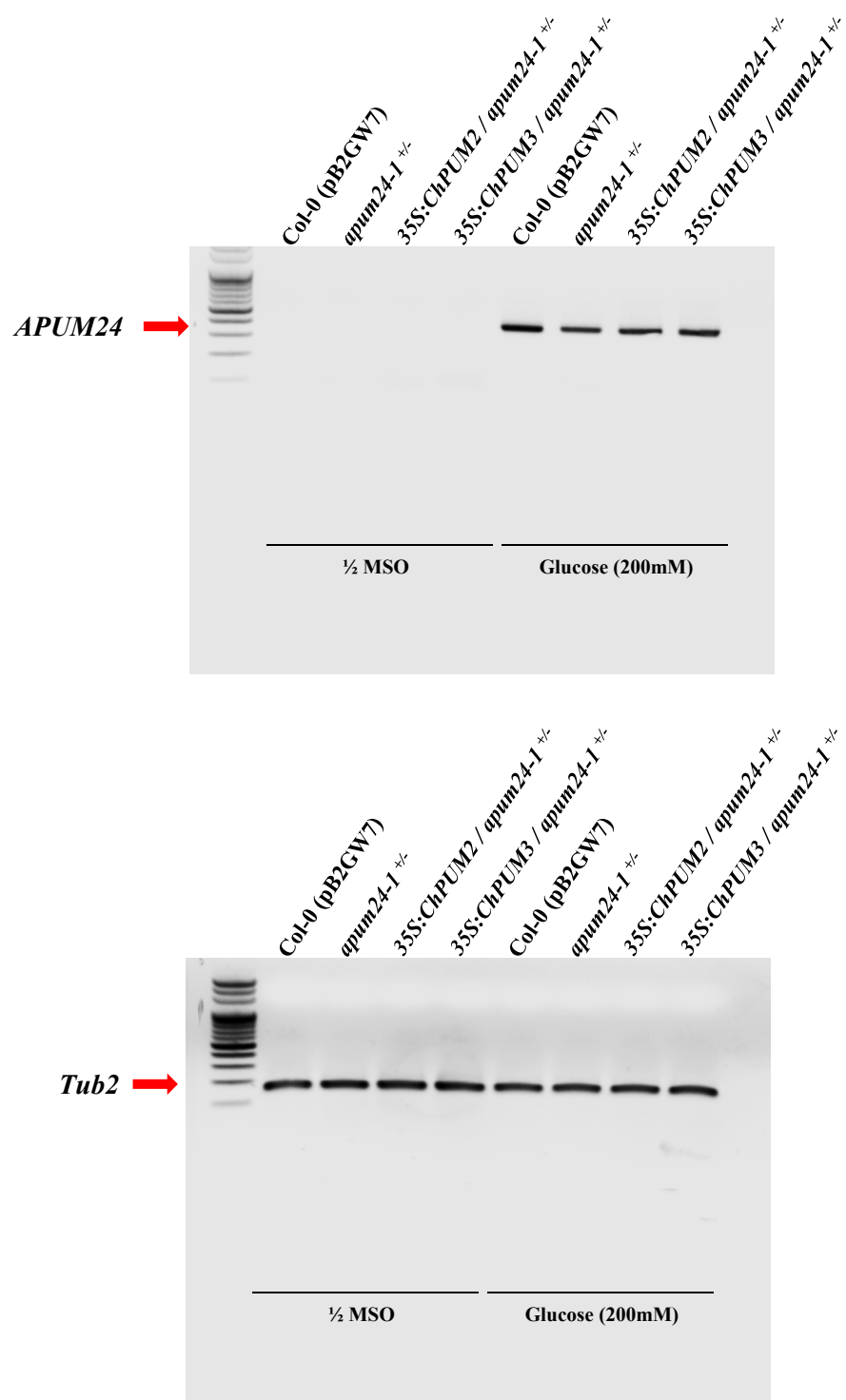

**Figure S5.** Original agarose gel images of RT-PCR products for Fig. 7b.

Supplement: Supplementary file 5 — Additional file 5: Figure S5. Original agarose gel images of RT-PCR products for Fig. 7b. [file 12870_2020_2444_MOESM5_ESM.pdf]
