# Supplementary material for: Structural and functional similarities and differences in nucleolar Pumilio RNA-binding proteins between Arabidopsis and the charophyte Chara corallina
Source: BMC Plant Biol. 2020 May 24;20:230. doi: 10.1186/s12870-020-02444-x (PMC7247198; doi:10.1186/s12870-020-02444-x)
Supplement: Supplementary file 4 — Additional file 4: Figure S4. Original agarose gel images of RT-PCR products for Fig. 6a and b. [file 12870_2020_2444_MOESM4_ESM.pdf]

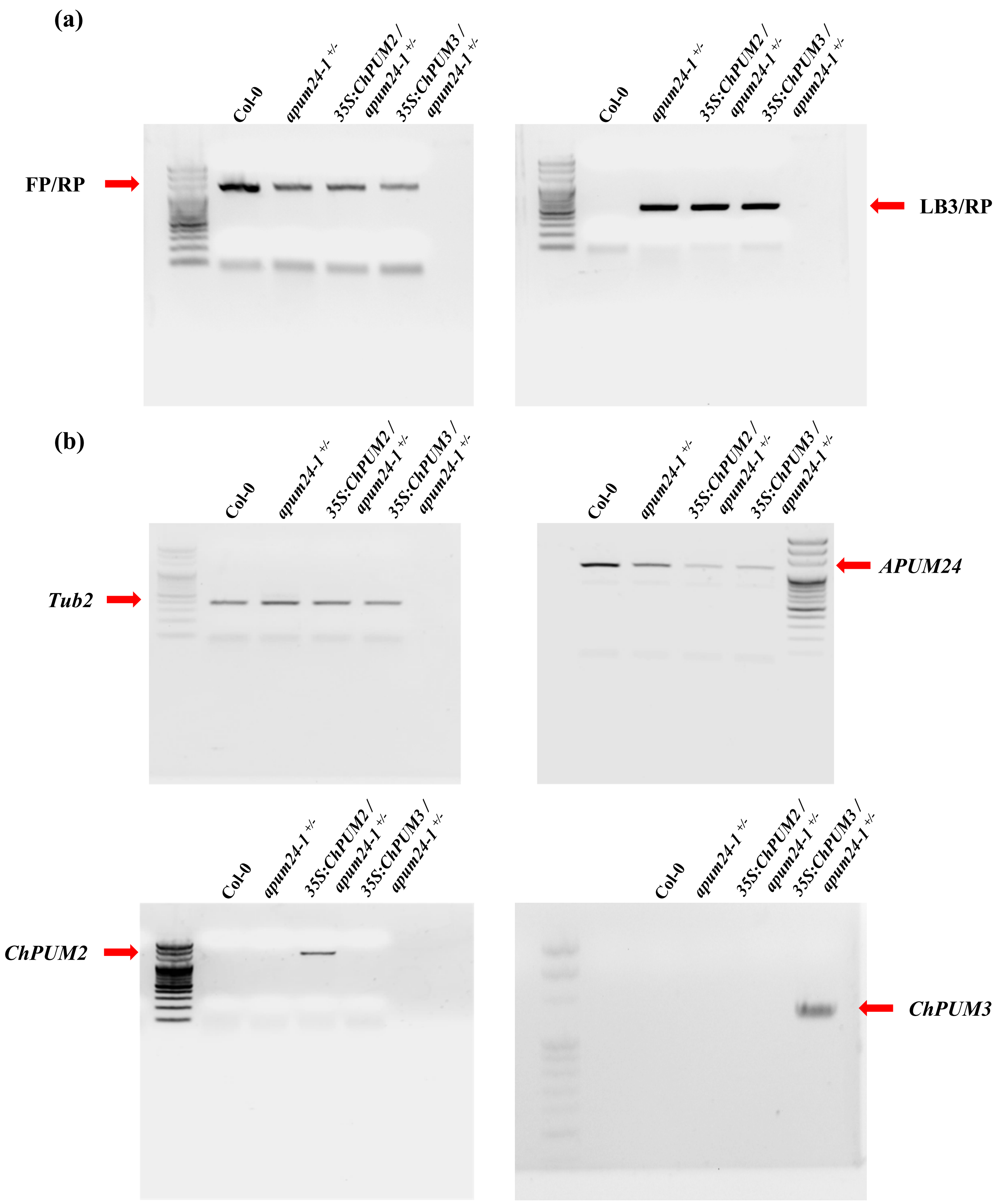

Figure S4. Original agarose gel images of RT-PCR products for Fig. 6a and 6b. (a) Genotyping images used in the bottom panel in Fig. 6a. Primer indicated with arrows are in the upper panel of Fig. 6a. (b) Original figures used in Fig. 6b.
