# Supplementary material for: Structural and functional similarities and differences in nucleolar Pumilio RNA-binding proteins between Arabidopsis and the charophyte Chara corallina
Source: BMC Plant Biol. 2020 May 24;20:230. doi: 10.1186/s12870-020-02444-x (PMC7247198; doi:10.1186/s12870-020-02444-x)
Supplement: Supplementary file 6 — Additional file 6: Figure S6. Alignment of 18S rRNA sequences of A. thaliana and C. corallina. The red box indicates the Arabidopsis rRNA sequence at nt positions 1141–1151 to which APUM23 binds, and the blue box shows the identical sequence at nt positions 1148–1158 of 18S rRNA in C. corallina. [file 12870_2020_2444_MOESM6_ESM.pdf]

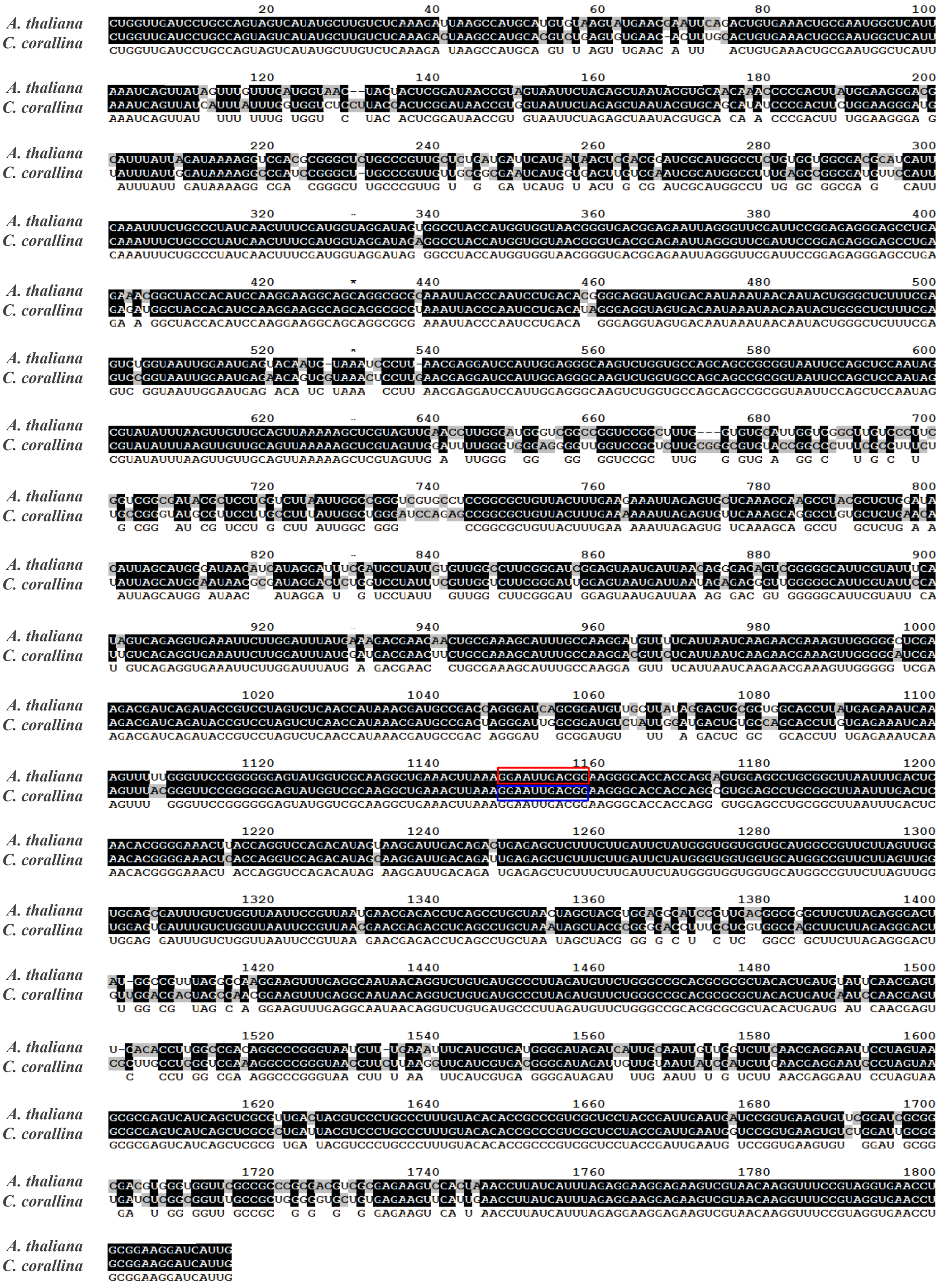


**Figure S6.** Alignment of 18S rRNA sequences of *A. thaliana* and *C. corallina*. The red box indicates the Arabidopsis rRNA sequence at nt positions 1141-1151 to which APUM23 binds, and the blue box shows the identical sequence at nt positions 1148-1158 of 18S rRNA in *C. corallina*.
