# Supplementary material for: Structural and functional similarities and differences in nucleolar Pumilio RNA-binding proteins between Arabidopsis and the charophyte Chara corallina
Source: BMC Plant Biol. 2020 May 24;20:230. doi: 10.1186/s12870-020-02444-x (PMC7247198; doi:10.1186/s12870-020-02444-x)
Supplement: Supplementary file 7 — Additional file 7: Figure S7. Alignment of 5.8S rRNA and ITS2 sequences of A. thaliana and C. corallina. (a) Alignment of 5.8S rRNA sequences. (b) Alignment of ITS2 sequences. [file 12870_2020_2444_MOESM7_ESM.pdf]

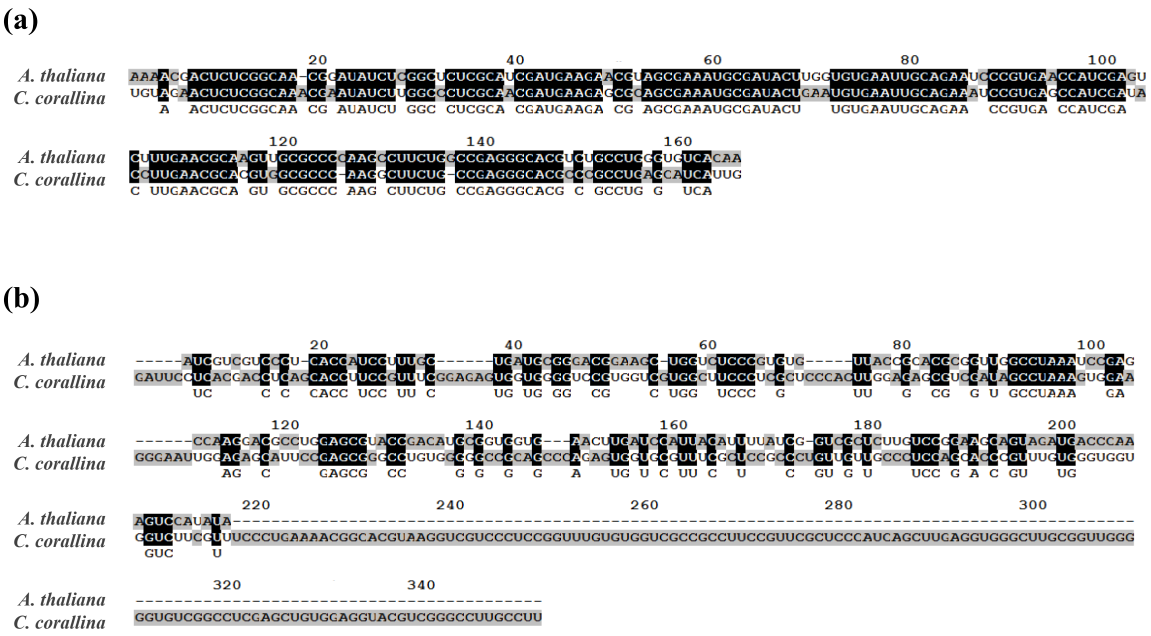

Figure S7. Alignment of 5.8S rRNA and ITS2 sequences of *A. thaliana* and *C. corallina.* (a) Alignment of 5.8S rRNA sequences. (b) Alignment of ITS2 sequences.
