# Supplementary material for: Structural and functional similarities and differences in nucleolar Pumilio RNA-binding proteins between Arabidopsis and the charophyte Chara corallina
Source: BMC Plant Biol. 2020 May 24;20:230. doi: 10.1186/s12870-020-02444-x (PMC7247198; doi:10.1186/s12870-020-02444-x)
Supplement: Supplementary file 8 — Additional file 8: Table S1. Primers used in this study. [file 12870_2020_2444_MOESM8_ESM.pdf]

**Table S1.** Primers used in this study

| primer name | sequence(5'→3') | reaction |
|---|---|---|
| ChPUM2-F | CACCATGGGGAAGAAGGGGCGAG | cloning |
| ChPUM2-R1 | CTACGACATCACAAACTGTGG | |
| ChPUM2-R2 | CGACATCACAAACTGTGG | |
| ChPUM3-F | CACCATGAGGATGAAGCCAAGG | |
| ChPUM3-R1 | TCATGACGGGGTGCCATCTCC | |
| ChPUM3-R2 | TGACGGGGTGCCATCTCCATC | |
| APUM23-F | CACCATGGGTGAACGAGGAAAGTC | |
| APUM23-R | AATTCTCATTTTATTTGAAT | |
| APUM24-F | CACCATGTTGTGTGTGTTTAGAAGGAAG | |
| APUM24-R | TGAAGACATCAGATTTCCTAGTTT | |
| 5'ETS-18S-F | AGATGACGGTCAAGACCTCG | qRT-PCR |
| 5'ETS-18S-R | CATTCGCAGTTTCACAGTCTG | |
| 18S-ITS1-F | GAATGATCCGGTGAAGTGTTCG | |
| 18S-ITS1-R | GAGAGTGGTGATCTTTGGTTCG | |
| 5.8S-ITS2-F | CCGTGAACCATCGAGTCTTTG | |
| 5.8S-ITS2 -R | CGTCCTTGGCTCGGATTTAG | |
| Tubulin4-F | GCTCGCTAATCCTACCTTTGG | |
| Tubulin4-R | AGCCTTGGGAATGGGATAAG | |
| FP | CATGGAAGCGCTAAGGTACAC | genotyping |
| RP | TTGTTCATCCTTCGTCTCCTC | |
| LB3 | TAGCATCTGAATTTCATAACCAATCTCGATACAC | |
| APUM23-RT-F | GAGTCTGCTCTCAAATCC | RT-PCR |
| APUM23-RT-R | GAATCTTTTGCCGTTTCAGTG | |
| APUM24-RT-F | GGAGCATCTAAGCAGCAG | |
| APUM24-RT-R | CACAGCTTCTCGCTCAGAG | |
| ChPUM2-RT-F | AGCTCCTTTGCTGGCCTG | |
| ChPUM2-RT-R | CTGCTGCATCTCATGTCCTG | |
| ChPUM3-RT-F | TAACAAGATGCTCGTTCAGGGTAATG | |
| ChPUM3-RT-R | GACCGCCGTTTGCCACCTC | |
| Tubulin2-RT-F | TTCCAGGTTTGTCACTCGTTG | |
| Tubulin2-RT-R | GCTTTCGGAGGTCAGAGTTG | |
| APUM24-RT-F2 | CATACCATCTGGGAAGTGCAGCAC | RT-PCR (for Fig.7b) |
| APUM24-RT-R2 | TGGCAAGCCTAGAACCATCACG | |
| Tubulin2-RT-F2 | GAGTACCCAGATCGCATGATGCTTAC | |
| Tubulin2-RT-R2 | GGTAGTGAGTTTGAGAGTCCTGAAGC | |
